# Clinical utility and dilemmas of SNP microarray testing

**DOI:** 10.1186/1755-8166-7-S1-I34

**Published:** 2014-01-21

**Authors:** Virginia Kimonis

**Affiliations:** 1 Division of Genetics and Genomics, Department of Pediatrics, University of California, Irvine, CA 92868

## 

The ability to diagnose patients with developmental delay, intellectual disability, congenital anomalies, and dysmorphic features has significantly improved with the introduction of SNP microarray technologies which includes more than 1.8 million markers on a single array. This high-density array will allow for sensitively detecting all known abnormalities with defined loci of interest as well as the discovery of ‘‘new syndromes’’ as a cause of mental retardation or autism. The most common micro deletion disorders include15q11-q13 Prader-Willi/Angelman, 22q11.3 velo-cardiofacial, 17pl3.3 William, 1p36and 16p11.2 microdeletion syndromes. SNP arrays are able to detect segmental uniparental disomy (UPD) as in Prader Willi syndrome and UPD14. SNP arrays identify parental consanguinity which may otherwise go undetected. Long stretches of homozygosity can be analyzed for recessive genes in patients born to consanguineous parents. (http://www.ccs.miami.edu/cgi-bin/ROH/ROH_analysis_tool.cgi)

Arrays cannot however identify balanced chromosomal rearrangements, such as translocations or inversions, Marker chromosomes may also be missed, depending on the size, marker composition, and array coverage of the specific chromosomal region present on the marker. Dilemmas arise when ‘‘CNVs of uncertain clinical significance’’ are identified, or if a parent is not available for testing, thus making it difficult to identify if the rearrangement is inherited or arose de-novo. Further dilemmas arise if a parent is identified with the same rearrangement and is apparently asymptomatic, should one then consider the presence of a mutation in a recessive gene in the other allele to explain autism, mental retardation or other disorders.

Overall SNP array technology has become the test of choice, permitting a 5.9% detection rate in patients with negative microarray-based CGH and an overall detection rate approaching 29%.

